# Update on the epidemiology of healthcare-acquired bacterial infections: focus on complicated skin and skin structure infections

**DOI:** 10.1093/jac/dkab350

**Published:** 2021-11-21

**Authors:** Mark H Wilcox, Matthew Dryden

**Affiliations:** 1 Department of Medical Microbiology, Leeds Teaching Hospitals & University of Leeds, Leeds, UK; 2 Hampshire Hospitals NHS Foundation Trust, Winchester, UK

## Abstract

Healthcare-associated infections (HCAIs) are a threat to patient safety and cause substantial medical and economic burden in acute care and long-term care facilities. Risk factors for HCAIs include patient characteristics, the type of care and the setting. Local surveillance data and microbiological characterization are crucial tools for guiding antimicrobial treatment and informing efforts to reduce the incidence of HCAI. Skin and soft tissue infections, including superficial and deep incisional surgical site infections, are among the most frequent HCAIs. Other skin and soft tissue infections associated with healthcare settings include vascular access site infections, infected burns and traumas, and decubitus ulcer infections.

## Introduction

Healthcare-associated infections (HCAIs) are infections acquired in any healthcare setting that were not present before the contact.^1^ They may result in (or prolong) stays in acute care hospitals, cause morbidity and mortality and increase medical and economic burdens. In England, there are an estimated 834 000 HCAIs per year among adult inpatients in National Health Service hospitals, accounting for 7.1 million occupied hospital bed days (21% of total annual bed days) and an annual economic burden of £2.7 billion.^2^ HCAIs are also an indicator of the quality of care.^3^ Causative organisms include both Gram-positive and Gram-negative pathogens, and some of these may be resistant to commonly used antimicrobial agents. The incidence of infections with antimicrobial-resistant pathogens has been increasing worldwide, with substantial associated additional health and economic burden.^4^ All these factors make reducing the rate of HCAIs a high priority.^5–7^

The risk of HCAIs is increased by patient characteristics such as immunosuppression, diabetes mellitus, cardiovascular disease, advanced age, and colonization with specific pathogens.^8–12^ Certain procedures, clinical settings and other factors are associated with a higher incidence of HCAI, including the ICU, invasive procedures and devices, recent antimicrobial use, the type and duration of surgery, and the length of stay (LOS).^8^ Surgical site infections (SSIs), a subset of skin and soft tissue infections (SSTIs), are among the most common HCAIs.^13–15^

## HCAI epidemiology

Real-time/continuous surveillance plays a major role in reducing HCAI frequency and the associated burden by providing prompt feedback so that problems can be addressed in multidisciplinary team meetings at the earliest opportunity. The gold standard for surveillance is continuous monitoring of incidence using standardized definitions and protocols, as in the SSI surveillance programme for National Health Service hospitals and treatment centres in England.^16^ Such programmes may focus on particular species or procedures and provide comprehensive data on incidence rates, trends in causative organisms, and antimicrobial resistance. This detailed information on patient- and surgery-related characteristics allows the establishment of benchmarks and the identification of risk factors. Most national HCAI surveillance programmes measure the incidence of a set of common HCAI types continuously,^17^ while more populous countries and international agencies may use point prevalence surveys that assess HCAIs in representative sentinel hospitals at specific timepoints. Detailed local surveillance data on individual surgery types provides useful feedback in the routine clinical setting, while the use of internationally recognized definitions and protocols allows comparison with other centres and published studies.^18^^,^^19^

### Acute care facilities

The first full ECDC point prevalence survey conducted in 2011–12 revealed an estimated HCAI prevalence in Europe of 6.0% (country range 2.3%–10.8%).^18^ SSTIs including SSIs were the most frequent infections (Table 1). The HCAI prevalence was highest among patients in ICUs (19.5%). Gram-negative organisms were identified in 52% of HCAIs with microbiology results, and *Escherichia coli* was the most common pathogen identified overall (Table 1). The microorganisms isolated most frequently from SSTIs were *Staphylococcus aureus* (29.2%), *Pseudomonas aeruginosa* (11.9%), *E. coli* (8.9%) and *Enterococcus faecalis* (7.4%).^20^

**Table 1.** Healthcare-associated infections in European acute care hospitals

Characteristics	Frequency in the ECDC Point Prevalence Survey (%)	
	2011–12^18^	2016–17^21^
HCAI type		
SSTI, including SSI	23.6	22.5
Lower respiratory tract	23.5	25.7
Urinary tract	19.0	18.9
Bloodstream	10.6	10.8
Gastrointestinal	7.6	8.8
Microorganisms isolated		
* Escherichia coli*	15.9	16.1
* Staphylococcus aureus*	12.3	11.6
* Enterococcus* spp.	9.6	9.7
* Pseudomonas aeruginosa*	8.9	8.0
* Klebsiella* spp.	8.7	10.4
Coagulase-negative staphylococci	7.5	7.1
* Clostridioides difficile*	5.4	7.3

ECDC, European Centre for Disease Prevention and Control; HCAI, healthcare-associated infections; SSTI, skin and soft tissue infection; SSI, surgical site infection.

A second ECDC survey conducted in 2016–17 revealed an estimated HCAI prevalence of 5.9% (country range 2.9%–10.0%); prevalence was again highest in ICUs (19.2%). The most frequent infections were lower respiratory tract infections, including pneumonia, which accounted for about one-quarter of HCAIs (Table 1). Most of the identified organisms were Gram-negative bacteria (52.1%), and *E. coli* was again the most commonly identified organism.^21^

The 2018 US CDC report on HCAI prevalence covered six common HCAIs [central line-associated bacteraemia, catheter-associated urinary tract infections (UTIs), ventilator-associated infections, SSIs, MRSA bacteraemia, and HCAIs involving *Clostridioides difficile*]*.* There was a 9% decrease from the previous year in bacteraemia associated with a central line, an 8% decrease in UTIs associated with catheters and a 12% decrease in nosocomial *C. difficile* infections; there were no significant changes in the rates of SSIs, MRSA bacteraemia, or ventilator-associated infections.^22^

In China, a point prevalence survey conducted in Dong Guan City in 2015 revealed a pooled HCAI prevalence in secondary and tertiary care settings of 2.9% (95% CI 2.6–3.1), with lower respiratory tract infection (LRTI) being the most frequently diagnosed HCAI (35.5%), followed by UTIs (17.0%), and SSIs (15.1%). Gram-negative bacteria were the most frequently isolated pathogens, accounting for 68.1%, and including *E. coli* (14.8%), *P. aeruginosa* (13.9%), *Klebsiella pneumoniae* (11.1%), and *Acinetobacter baumannii* (10.9%).^23^*S. aureus* was the pathogen isolated most frequently from SSTIs (21.7%); however, Gram-negative pathogens accounted for most infections, with *P. aeruginosa* (15.2%) and *A. baumannii* (10.9%) being the most common.^23^ A 2014 point prevalence survey in 124 hospitals in Beijing province revealed an HCAI prevalence of 2.2%.^24^ Respiratory tract infections were the most common HCAIs (54.4% overall; lower tract 46.7%, upper tract 6.8%), followed by UTIs (15.0%), gastrointestinal tract infections (7.7%), SSIs (6.3%) and bloodstream infections (5.5%). Also here, the most frequently isolated pathogens were Gram-negative organisms, including *P. aeruginosa, A. baumannii* and *E. coli*. The HCAI prevalence was 14.5% among patients in ICU.^24^ Another point prevalence survey conducted in 2014/2015 in 52 hospitals located in 22 Chinese provinces revealed a prevalence of 3.7%, with the most common infections being LRTIs (47.2%), followed by UTIs (12.3%), upper respiratory tract infections (11.0%), and SSTIs including superficial and deep incisional SSIs (10.8%).^25^ The HCAI rate was highest in the ICU (17.1%). Gram-negative pathogens were isolated most frequently overall (*P. aeruginosa* 9.4%, *A. baumannii* 7.9%, and *K. pneumoniae* 7.3%), while *S. aureus* was the most frequent pathogen isolated from SSTIs (23.0%), followed by *P. aeruginosa* (14.2%), and *A. baumannii* (7.1%).^25^

### Long-term care facilities

Long-term care facilities (LTCF) comprise a heterogenous setting. Most of them provide residential skilled nursing care for the elderly, but a variety of facilities are included that provide long-term care for residents with mental or physical disabilities, regardless of age. In 2013, the first ECDC point prevalence survey in European LTCFs revealed a crude HCAI prevalence of 3.4% (country range: 0.4%–7.1%), corresponding to an estimated 4.2 million HCAIs per year.^26^ The most frequent infections were respiratory tract infection (31.2%), UTIs (31.2%) and skin infections (22.8%). Most skin infections (87.4%) were described as cellulitis, soft tissue, or wound infections.^26^ The most common pathogens were *E. coli* (34.4%), *S. aureus* (10.2%), and *Proteus mirabilis* (8.1%); however, there was great variation in reporting of microbiological data by country and the results should be interpreted with caution.

A subsequent European LTCF survey conducted between 2016–17 revealed a prevalence of 3.7% (country range: 0.9%–8.5%), corresponding to an estimated 4.4 million cases (95% CI: 2.0–8.0 million). The most frequent types of HCAI in LTCF were similar to the previous survey: respiratory tract infections (33.2%), UTIs (32.0%) and skin infections (21.5%).^21^ In a subset of centres with sufficient data, the combined prevalence of MRSA, vancomycin-resistant *Enterococcus faecalis*, Enterobacterales resistant to third-generation cephalosporins, and carbapenem-resistant *P. aeruginosa* and *A. baumannii* was 28.0%. This survey was conducted during the same period as the 2016–17 European acute care hospital point prevalence survey, and the results were reported together.^21^ The estimated annual burden of SSTIs in LTCFs was five times higher than that in acute care hospitals at 626 415 [cumulative 95% CI (cCI 95%) 388 293–896 687] versus 108 269 (cCI 95% 45 149–242 816). This suggests that LTCF may be a ‘reservoir’ for antimicrobial resistance and that more stewardship efforts are needed in this setting.

## Focus on healthcare-acquired complicated skin and soft tissue infections, including incisional surgical site infections

SSTIs are among the most common HCAIs and are frequent reasons for extended hospitalization and re-admission. SSTIs types that may be acquired in healthcare settings are listed in Table 2. Many of these are commonly acquired in the community setting, and the timing of their onset is a key component of case definitions for HCAIs. Their clinical presentation ranges from mild forms of erysipelas or cellulitis to rare, life-threatening necrotizing soft tissue infections that can lead rapidly to sepsis with multisystem organ failure. This wide range of disease severities can lead to over-treatment of uncomplicated infections. Complicated skin and soft tissue infections (cSSTIs) require hospitalization.^13^^,^^27–29^

**Table 2.** Skin and soft tissue infections that may be associated with a healthcare setting

Healthcare-associated SSTI
Surgical site infection
Vascular access site infection
Cellulitis
Decubitus ulcer infection
Diabetic foot infection
Infected burns and traumas
Breast abscesses or mastitis
Amputation stump infection
Newborn circumcision infection
Omphalitis in a newborn

In 2013, the US FDA established a uniform description of a subset of complicated SSTIs that require treatment as ‘Acute Bacterial Skin and Skin Structure Infections’ (ABSSSIs), which includes cellulitis, erysipelas, wound infections, and major cutaneous abscesses with a lesion surface area ≥75 cm^2^, but excludes infections requiring complex treatment regimens, such as necrotizing soft tissue infections, diabetic foot infections, and decubitus ulcers.^30^ ABSSSIs represent a substantial burden to the healthcare system.^27^^,^^31^ The FDA combined this description with quantifiable criteria for assessing early clinical response (after 48–72 h of treatment) to be used as the primary endpoint in trials of new antimicrobials for skin infections.^30^ Because it would not be ethical to randomize patients with these infections to placebo, the FDA introduced these changes to overcome issues with non-inferiority trial designs due to the possible spontaneous improvement of some infections. Meanwhile, the EMA requires that clinical trials have primary efficacy endpoints based on assessment at test-of-cure, conducted a specified number of days after treatment is completed to assess resolution of the infection and the clinical outcome.^32^ A post hoc analysis of data from the Phase 3 registration trials for tedizolid indicated that early response to therapy predicts late treatment success; however, a lack of early response did not predict late treatment failure.^33^ Analysis of data from the REACH study indicated that lack of early response was associated with adverse clinical outcomes, higher use of healthcare resources and more frequent treatment modification.^34^ Inappropriate initial treatment is a major determinant of poor clinical and economic outcomes.^35–37^

## Surgical site infections

The most common cSSTIs acquired in the acute care setting are superficial and deep incisional SSIs. SSIs involving organ and body space are not considered cSSTIs. The distribution of SSI types differs by surgical procedure. Recent surveillance data from Public Health England (PHE) reveal that superficial and deep incisional infections account for 70% to 90% of SSIs detected in hospital or on readmission after the most common major surgical procedures, apart from bile duct, liver, or pancreatic procedures where organ/space infections were more common (75%).^12^

Criteria for defining SSIs have been established based on the CDC definitions for superficial and deep incisional infections.^38^ Superficial incisional SSIs include infections involving only skin and/or subcutaneous tissue that occur within 30 days after surgery and are accompanied by purulent drainage or microbiological confirmation, and typical signs and symptoms of infection. Deep incisional SSIs are infections involving deep soft tissues (e.g. fascia and/or muscle) occurring within 30 days after surgery (up to 1 year if prosthetic material is implanted) and associated with purulent drainage, or wound opening with typical signs and symptoms of infection, an abscess or other evidence of infection identified through direct examination, reoperation, or through histopathologic or radiologic tests.^38^ Most surveillance programmes employ these criteria or modifications thereof. PHE’s SSI surveillance programme uses the US CDC definitions and also assesses leucocytes in infections that have been identified microbiologically, in the absence of obvious clinical signs/symptoms, and requires ≥2 clinical signs/symptoms of infection for superficial incisional SSIs.^12^

A systematic review of the economic and health burden of SSI in six European countries (France, Germany, Italy, the Netherlands, Spain and the UK) confirmed that SSIs represent a substantial financial burden, with the majority of extra costs attributable to prolonged LOS.^39^ Hospital discharge records in the US revealed that SSIs increased LOS by 13.7 days after cardiovascular surgery and 5.7 days after skin, subcutaneous tissue, and breast surgeries.^40^ Meta-analysis of the financial impact of HCAIs on the US health-care system showed that SSIs are the most costly, contributing about one-third of the overall costs associated with the five most-frequent HCAIs.^41^

In addition to their economic burden, SSIs increase mortality and cause personal distress, loss of work productivity and reduced quality of life. Complicated SSTIs are more common among people with comorbid conditions, such as diabetes, obesity, critical illness, immunosuppression, liver and kidney disease, and vascular insufficiency.^29^^,^^42^^,^^43^ Worldwide, the SSI burden is higher in resource-limited countries.^44–47^ SSI surveillance programmes are less well-established in these countries.^48^ The WHO has published global guidelines on SSI prevention that cover pre-, intra- and post-operative periods, and stress the importance of SSI surveillance.^47^

The International Nosocomial Infection Control Consortium (INICC) conducts outcome surveillance of SSI incidence rates in patients undergoing surgery in 30 countries in Latin America, Asia, Africa, and Europe, stratified by procedure type.^49^ Between 2005 and 2010, the consortium collected data on 260 973 surgeries, which resulted in 7523 SSIs (2.9%). Procedures with the highest SSI rates were ventricular shunts (12.9%), colon surgery (9.4%), and bile duct, liver, or pancreatic surgery (9.2%); the lowest rates were for thyroid surgeries (0.3%) and caesarean deliveries (0.7%).^49^

Based on point prevalence survey data from 2011–12, the estimated burden of SSIs in the EU/EEA was nearly 800 000 [95% uncertainty interval (UI) 762 721–835 448] cases per year.^50^ In 2016–17, the ECDC estimated that the annual SSI burden Europe was lower, at 518 182 (95% UI 293 036–858 222) SSI per year.^21^ Estimation of SSI rates is complicated by the fact that they often become evident after the patient has been discharged.^51^^,^^52^ National surveillance data from Italy shows that up to 60% of SSIs were discovered at 30 day post discharge surveillance.^53^ Local SSI surveillance reports are the most relevant feedback for surgeons.^54^ They should include comparison with other centres or published data to put findings in perspective.

## Microbiology

It is not always possible to culture pathogens from skin infections; however, culturing should always be attempted in complicated infections.^55^ Superficial swabbing may detect colonizing bacteria that are not involved in the infection; aspirates or biopsy from the leading edge of the infection are more useful.^56^ Sensitive molecular testing conducted directly on clinical samples without culturing may help to identify pathogens rapidly and provide some indications of drug susceptibility.^57^

The most common pathogens in many settings are Gram-positive organisms such as *S. aureus,* and β-haemolytic streptococci.^28^^,^^58^^,^^59^ Coagulase-negative staphylococci are generally not considered pathogens in skin infections that do not involve foreign/prosthetic material. Their isolation from skin infection samples is generally either not reported or attributed to contamination; however, repeated isolation as the sole microbe from a skin infection site may be an indication of a pathogenic role. Gram-negative organisms have become significant causes of SSTIs in both healthcare-associated and community-associated infections.^12^^,^^28^^,^^59–61^

Pathogens causing SSIs are usually commensal bacteria associated with the surgical site, including bacteria from the normal skin flora or endogenous microflora from internal organs.^62^^,^^63^ Gram-negative pathogens are more frequent in SSIs occurring after abdominal surgery.^64^ Resistant pathogens include Gram-positive MRSA and VRE, but in some settings MDR Gram-negative organisms are common and nosocomial transmission of these pathogens is well described.^65–67^ Continuous surveillance data from the PHE SSI surveillance programme revealed a clear decrease in the involvement of *S. aureus* in SSI and a proportional increase in Gram-negative pathogens over the past 15 years.^12^^,^^68^ Enterobacterales now represent the largest proportion of pathogens and most of the *S. aureus* is MSSA.^12^

## SSTI management

The first step in managing patients who present with a skin infection is to quickly identify those who are likely to have or develop rare necrotizing soft tissue infections and treat them according to current guidelines.^55^^,^^69^ Patients with cSSTIs will need antimicrobial therapy and/or surgical drainage or debridement. Risk stratification of patients should follow an established approach and consider MRSA coverage depending on local surveillance data and risk factors.

The selection of initial antimicrobial therapy is generally empirical and must consider local epidemiology, the prevalence of resistant pathogens, the site and severity of the infection, patient characteristics, and clinical complications. A substantial portion of patients receive initial empirical antimicrobial treatment that is inadequate or inappropriate for the pathogen, resulting in poorer outcomes and increased resource use.^28^^,^^35–37^^,^^70^ In the retrospective, observational REACH study, adults with complicated SSTI (*n = *1995) treated in 10 European countries had a mean LOS of 18.5 days; but this increased by 10.9 days in patients needing a change of initial antimicrobial treatment.^36^ Infections with resistant pathogens and mixed infections caused by both Gram-positive and Gram-negative pathogens are associated with a higher risk of inappropriate empirical treatment.^37^

Uncertainty regarding the appropriate empirical treatment to administer, combined with the potential for rapidly changing epidemiology, drives the choice of treatment toward a broad-spectrum agent; however, overuse of these agents can lead to the development of resistance. Antimicrobial stewardship principles stress the need for pathogen identification, reassessment, and treatment de-escalation to narrow-spectrum agents as soon as possible.^71^

The most frequently reported risk factors for MRSA in cSSTI include recent antibiotic use, colonization or previous infection by MRSA, hospitalization within the previous year, history of IV drug use, end-stage renal disease and/or haemodialysis, presence of an abscess, obesity, and a history of HIV or diabetes.^72^ Knowledge of risk factors for antimicrobial-resistant SSTIs (Table 3) can help to guide the initial choice of antimicrobial therapy.^73^ Reported risk factors for cSSTIs caused by mixed pathogens include admission to an ICU, having an infection other than an abscess, and living in an LTCF.^74^

**Table 3.** Main predictors and clinical presentation for skin and soft tissue infections due to multidrug-resistant bacteria^a^

Pathogen	Main predisposing factors^b^	Usual clinical presentations
CA-MRSA	Close contact/communal living (e.g. prisoners, athletes, military recruits, MSM, IVDU). Frailty (e.g. homeless people or residents of elderly home). Travel in high-prevalence area.	Furunculosis, skin abscess, cellulitis or NF (uncommon).
HA-MRSA	Nasal and/or skin carriage, old age, haemodialysis, recent or extended hospital stay, invasive procedures, antimicrobial exposure, ICU admission, immunosuppression, living in LTCF, receiving home nursing care, chronic skin lesions, prior MRSA infection.	Cellulitis related to chronic wound or ulcer, DFI and SWI.
*Pseudomonas aeruginosa*	Extended hospital stays, invasive procedures, antimicrobial exposure, ICU admission, immunosuppression, prior *P. aeruginosa* infection.	Cellulitis related to chronic wound or ulcer, DFI, SWI, NF (uncommon) and ecthyma gangrenosum (neutropenia).
ESBLE and CPE	Extended hospital stays, invasive procedures, antimicrobial exposure, ICU admission, intestinal colonization, recent travel in endemic regions.	Cellulitis related to chronic wound or ulcer, DFI, SWI and NF (uncommon).
*Acinetobacter baumannii*	Extended hospital stays, invasive procedures, antimicrobial exposure, ICU admission, warm weather.	Cellulitis related to chronic wound or ulcer, DFI and community-acquired trauma-related SSTI.
VRE	Extended hospital stays, invasive procedures, antimicrobial exposure, ICU admission, intestinal colonization.	DFI and SWI
*Aeromonas hydrophila*	Microtrauma or wound contamination by fresh or brackish water.	NF
*Vibrio vulnificus*	Microtrauma or wound contamination by seawater or seafood.	NF

CA-MRSA, community-associated methicillin-resistant *Staphylococcus aureus*; CPE, carbapenemase-producing Enterobacterales; DFI, diabetic foot infection; ESBLE, extended-spectrum β-lactamase-producing Enterobacterales; HA-MRSA, hospital-acquired MRSA; IVDU, intravenous drug user; LTCF, long-term care facility; MSM, men who have sex with men; NF, necrotizing fasciitis; SSTI, skin and soft tissue infection; SWI, surgical wound infection; VRE, vancomycin-resistant enterococci.

a This Table is modified, with permission, from Table 1 in Barbier *et al*.^73^

b Local epidemiological patterns (i.e. endemicity or active outbreak of a given pathogen) must be considered for all healthcare-associated cases of SSTI.

### SSI management

**Figure 1.**
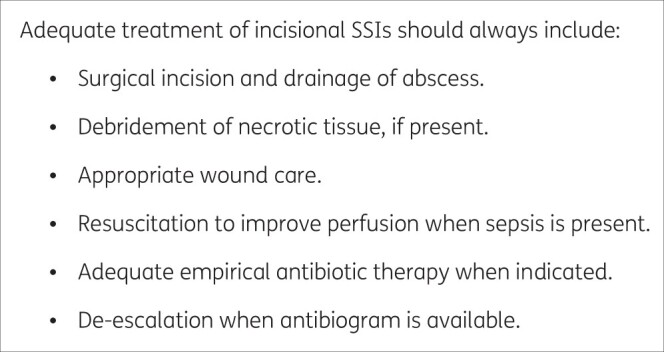
Key steps in SSI management from the 2018 WSES/SIS-E consensus conference on management of skin and soft-tissue infections.^69^

A surgical wound should be sampled for microbiological testing if there is suspicion of infection. The World Society of Emergency Surgery (WSES) and the Surgical Infection Society Europe (SIS-E) provide recommendation for managing incisional SSIs; key steps are presented in Figure 1. Please refer to the full guideline for details.^69^

Superficial SSIs are usually managed without antibiotics. Broad-spectrum antibiotic treatment that covers pathogens associated with the surgical procedure should be initiated empirically if there are signs of systemic inflammatory response or organ failure, and the choice of definitive treatment should be guided by the response to treatment and the microbiology report.^69^

## Summary

HCAIs significantly increase the medical and economic burdens associated with care. Surveillance programmes provide critical feedback to inform efforts to prevent or manage HCAIs. Risk factors include patient characteristics, the type of procedure and setting. Complicated SSTIs, including SSIs, are among the most frequent HCAIs and represent a substantial health burden. Although it is not always possible to identify the pathogens in SSTIs, microbiological characterization and local surveillance data are crucial tools for guiding antimicrobial treatment.

## Transparency declarations

M.H.W. has received honoraria for consultancy work, financial support to attend meetings and research funding from Bayer, Durata, Menarini, MotifBio, Nabriva Therapeutics plc, Paratek, Tetraphase Pharmaceuticals, Inc Pfizer and The Medicines Company. M.D. has received honoraria from and attended advisory boards for Pfizer, Matoke and Menarini. Richard Vernell prepared a first draft of this manuscript on behalf of Springer Healthcare Communications. This medical writing assistance was funded by A. Menarini Farmaceutica Internazionale.

This paper was published as part of a Supplement sponsored and financially supported by A. Menarini Farmaceutica Internazionale.
